# Meteorological Table

**Published:** 1811-02

**Authors:** 


### METEOROLOGICAL TABLE.

### *From Dec. 29, to Jan. 27.*

| | D | Therm. | | | Barom. | | | Hygrom. dry | | | damp | | | Weather. |
|---|---|---|---|---|---|---|---|---|---|---|---|---|---|---|
| | 29 | 34 | — | 35 | $30^{3}$ | — | —$^{4}$ | | | | 4 | 10 | 15 | cloud . . snow . fair . . . |
| | 30 | 33 | 35 | 34 | — | — | — | | | | 14 | 1 | 16 | fair . . . . — snow . cloud . . |
| | 31 | 32 | 34 | 32 | — | — | —$^{3}$ | | | | 14 | 12 | — | cloud . . . snow . cloud . . . . |
| ◐ | 1 | 32 | 31 | 30 | —$^{3}$ | —$^{2}$ | —$^{1}$ | | | | 10 | 15 | 9 | cloud ... snow... fair.. clou... |
| | 2 | — | 31 | 30 | — | $29^{9}$ | —$^{8}$ | | | | 9 | 4 | 11 | clou . . . . snow . snow . . . |
| | 3 | 29 | 27 | 25 | —$^{7}$ | — | —$^{8}$ | | | | 15 | 10 | 7 | clou . . . . snow . . — . SW |
| | 4 | 29 | 30 | 29 | — | —$^{7}$ | — | | | | 10 | — | 1 | clou .... snow. clou .... E... |
| | 5 | 27 | 29 | 28 | —$^{8}$ | — | — | 15 | 20 | 16 | | | | fair . . . — . . clou . . . E . . . |
| | 6 | 29 | — | 27 | — | — | — | 16 | — | 15 | | | | clou .... fair ... — .... NE.. |
| | 7 | 28 | 29 | 30 | — | — | — | 12 | — | 11 | | | | clou .... fair... clou .. NE.... |
| | 8 | 29 | 30 | 29 | — | — | — | 9 | — | 10 | | | | clou . . — — NE . . |
| ○ | 9 | 31 | 32 | — | — | — | — | | | | 1 | 10 | 4 | clou .... snow ... rain . NE.. |
| | 10 | 28 | 32 | 36 | $29^{9}$ | — | —$^{8}$ | | | | 20 | 22 | 35 | fog . . . . — ... rain.. inn. ... |
| | 11 | 42 | 43 | 38 | —$^{6}$ | —$^{7}$ | —$^{9}$ | | | | 15 | 32 | 16 | rain .. clou ... fair .... SW.. |
| | 12 | 42 | 46 | — | —$^{6}$ | —$^{5}$ | — | | | | 14 | 17 | 12 | clou . . . rain .. fair . SW. . . |
| | 13 | 40 | 46 | — | —$^{6}$ | — | — | 5 | 2 | — | — | — | 1 | fair.. rain.. —.. inn... SW.. |
| | 14 | 47 | 46 | — | — | — | — | | | | 10 | 13 | 9 | cloud . . rain . clou . . SW.. |
| | 15 | 45 | 40 | 37 | —$^{7}$ | — | — | | | | 8 | 9 | 20 | clou. — . . fair. . . . SW .. |
| | 16 | 36 | 40 | — | —$^{8}$ | 30 | — | | | | 32 | — | 30 | fair ... — .... — ... W .. |
| ◑ | 17 | 46 | 51 | 44 | $29^{8}$ | —$^{6}$ | —$^{7}$ | | | | 55 | 66 | 50 | rain .. cloud .... — ... W .. |
| | 18 | 42 | — | 37 | —$^{7}$ | — | 30 | | | | 50 | 45 | 29 | cloud .... fair .. — .... W.. |
| | 19 | 36 | 41 | 38 | —$^{3}$ | — | —$^{4}$ | | | | 30 | 28 | 38 | fair ... — .... — ... W..N.. |
| | 20 | 37 | 40 | 36 | —$^{3}$ | —$^{2}$ | —$^{1}$ | | | | 47 | 28 | 30 | fair ... — .... — .... W .. |
| | 21 | 37 | 40 | 30 | 30 | — | —$^{1}$ | | | | 34 | 40 | 38 | cloud ... rain . fog ... SW.. |
| | 22 | 35 | — | 39 | —$^{2}$ | — | — | | | | 52 | — | 48 | fog .... — ... — .. SW . |
| | 23 | 37 | 42 | 40 | —$^{2}$ | — | —$^{3}$ | | | | 48 | — | 52 | rain , fair ... cloud .... W . |
| ● | 24 | 38 | 40 | 39 | —$^{3}$ | — | —$^{4}$ | | | | 50 | 48 | 47 | fair ... — cloud . . . E . . |
| | 25 | 38 | 39 | 36 | — | — | — | | | | 45 | 38 | 36 | cloud ... — .. — ... NE . |
| | 26 | 35 | 40 | — | —$^{2}$ | 30 | $29^{8}$ | | | | 40 | 45 | 51 | fair . cloud . . . — . W . . |
| | 27 | 41 | 40 | 36 | —$^{6}$ | — | — | | | | 50 | 27 | 24 | rain .. fair ... cloud ... W . |

30th December. Snow covers the ground. Ice an inch and half thick.

Jan. 1. Heavy fall of snow in the night, and morning of the 2d.

3. Though wind at S W. snow falling, and the temperature sensibly warmer, the thermometer falls to 27, and in the evening to 25.

4, 5, 6, 7, and 8. Wind from N. and N. E. with a sensation of cold far beyond the degree indicated on the thermometer. Atmosphere very dry. Ice from four to six inches thick.

9. The approach of a change indicated by the hygrometer returning to the humid point; on the 10th, an entire break up of the frost with light rain followed by very dense fog.

13. When rain was falling, the hygrometer rises to the dry side of the scale; and on the 15th and 16th, when fair, shews considerable humidity.

*Prince's-Street, Cavendish-Square, Jan. 29,* 1811.

